# Supplementary material for: Dietary Live Yeast Supplementation Influence on Cow’s Milk, Teat and Bedding Microbiota in a Grass-Diet Dairy System
Source: Microorganisms. 2023 Mar 6;11(3):673. doi: 10.3390/microorganisms11030673 (PMC10053648; doi:10.3390/microorganisms11030673)
Supplement: Supplementary file 1 [file microorganisms-11-00673-s001.zip › microorganisms-2203644-supplementary.pdf]

Table S1. Composition and analytical values of the total mixed rations (TMR).

TMR	C[1]	LY[1]
**Ingredients** (kg DM/cow/d)		
1st cut haylage	6.5	6.5
2nd cut haylage	3.2	3.2
2 nd cut hay	7	7
Expert Gala Marcenat (energy balanced concentrate)	3.5	3.5
Gala Proteine Fromagere C (protein balanced concentrate)	1.8	
Gala Proteine Fromagere LY (protein balanced concentrate with live yeast)		1.8
Galaphos Midi Repro (minerals)	0.18	0.18
Analytical values (% DM unless specified otherwise)		
DM, %	66.2 ± 5.2	62.7 ± 7.5
Crude Protein (CP)	14.5 ± 1.3	14.5 ± 1.9
Soluble Protein., % CP	29.0 ± 3.8	31.4 ± 3.6
Fat	1.9 ± 0.3	1.6 ± 0.3
Ash	9.0 ± 0.5	9.2 ± 1.3
Neutral Detergent Fibre amylase treatment Organic Matter (aNDFom)	45.0 ± 3.7	44.9 ± 1.6
Neutral Detergent Fibre amylase treatment (aNDF)	47.9 ± 3.5	47.8 ± 1.5
Acid Detergent Fibre (ADF)	33.2 ± 2.7	33.5 ± 1.5
Acid Detergent Lignin (ADL)	5.8 ± 0.5	5.4 ± 0.6
Water Soluble Carbohydrate (WSC)	11.4 ± 0.9	11.0 ± 1.0
Starch	1.8 ± 1.8	1.8 ± 1.8
Non Fibre Carbohydrate (NFC), %	28.5 ± 3.0	28.7 ± 3.2
Net Energy for Lactation (NEL)), Mcal/kg	1.37 ± 0.05	1.35 ± 0.04
Feed Unit for Lactation (UFL), /kg DM*	0.778 ± 0.03	0.767 ± 0.02

* *UFL = NEL (kcal/kg) / 1760 (INRA 2018).* [1]TMR C = total mixed ration of the control group; TMR LY = total mixed ration of the group receiving the live yeast supplementation.

Table S2. Concentrates composition and values.

	Expert Gala Marcenat	Gala Proteine Fromagere
**Raw material**		
Maize	18.21	
Wheat	15	
Barley	15	
Fine bran	15	
Soybean meal		48.5
Sunflower meal	15	3
Sunflower meal, shelled		23
Rape seed meal	11.9	23.4

Cereal seed hulls	5	
Cane molasses	2	1
Calcium carbonate	1	0.15
Oligo-elements, vitamins	0.75	
Amiviv SE	0.5	
Turboviv SE		0.45
Salt	0.44	
Biotine	0.2	
**Nutrients**		
Feed Unit for Lactation (UFL)	0.98	0.96
Crude Protein (%)	38.9	15.3
Fat (%)	1.4	2.4
Ash (%)	7	6.5
Crude cellulose (%)	11.2	9.2
Starch (%)	3.5	34.2
Phosphorus (%)	0.8	0.6
Calcium (%)	0.7	0.9
Magnesium (%)	0.4	0.3
Copper (mg/kg)	20.8	26
Sulphur (mg/kg)	0.5	0.31
Manganese (mg/kg)	92.2	158
Iode (mg/kg)	0.2	0.91
Cobalt (mg/kg)	0.6	0.4
Selenium (mg/kg)	0.2	0.4
Iron (mg/kg)	292.1	207
Zinc (mg/kg)	115.6	196
Ruminally degradable nitrogen (PDIN, g/kg)	304	117
Metabolisable protein supply (PDIE, g/kg)	228	117

Table S3. Alpha-diversity indexes calculated from the bacterial profiles in the raw milk and the teat suspensions according to the group (C=control or LY= live yeast) and the period (P1 = before yeast supplementation; P2 = after three month of yeast supplementation; P3 = after four months of supplementation).

	Group				Time				
	C	Y	*P*-value	SEM	P1	P2	P3	*P*-value	SEM
Milk (n=116)									
Observed	61.9	63.6	0.7	4.2	71.7	67.6	49.0	<0.001	3.5
Shannon	2.5	2.5	0.9	0.3	2.7	2.7	2.2	<0.001	0.3
Teat (n=56)									
Observed	96.7	93.1	0.4	9.5	108.4	103	72	<0.001	6.5
Shannon	3.1	3.1	0.9	0.1	3.31	3.25	2.76	0.02	0.08

Table S4. *P*-values from PERMANOVA on Bray Curtis and generalized Unifrac indexes of the milk and the teat suspensions according to the group (C=control or LY=live yeast) and to the period (P1 = before yeast supplementation; P2 = after three month of yeast supplementation; P3 = after four months of supplementation).

	Bray Curtis		Unifrac	
	Group	Time	Group	Time
Fungi				
Milk (n=101)	0.07	0.001	0.05	0.001
Teat (n=55)	<0.01	0.001	<0.01	0.001
Bacteria				
Milk (n=116)	0.10	0.001	0.47	0.001
Teat (n=56)	0.13	0.001	0.13	0.001

Table S5. Fungal and bacterial Observed and Shannon indexes of individual (a) teat and (b) milk.

(a) Teat

			Fungi		Bacteria	
Cow	Period	Group	Observed	Shannon	Observed	Shannon
1624	P1	C	64	3,34	98	3,19
3651	P1	C	19	2,49	129	3,31
4155	P1	C	56	3,09	122	3,32
4601	P1	C	14	2,18	89	3,14
4624	P1	C	58	3,35	136	3,60
5657	P1	C	81	3,35	109	3,31
5683	P1	C	76	3,40	132	4,11
5702	P1	C	20	1,89	117	3,35
5741	P1	C	23	2,57	89	3,03
2158	P1	LY	64	3,46	116	3,52
2168	P1	LY	17	0,56	123	3,53
2635	P1	LY	17	2,05	113	3,41
3180	P1	LY	16	2,03	88	3,02
4600	P1	LY	67	3,38	107	3,11
5642	P1	LY	18	2,43	96	3,01
5681	P1	LY	39	3,17	89	3,46
5706	P1	LY	71	3,26	109	3,24
5724	P1	LY	23	2,72	114	3,07
5732	P1	LY	27	2,70	84	3,07
1624	P2	C	37	2,64	114	3,27
3651	P2	C	24	1,90	87	3,04
4155	P2	C	60	2,84	116	3,56
4601	P2	C	45	3,38	132	3,43
4624	P2	C	53	2,50	129	3,93
5657	P2	C	29	2,67	89	3,08
5683	P2	C	33	1,97	121	3,27

5702	P2	C	62	1,45	100	3,38
5741	P2	C	55	2,36	76	2,76
2158	P2	LY	37	3,06	72	3,05
2168	P2	LY	26	1,06	85	3,30
2635	P2	LY	42	3,05	138	3,46
3180	P2	LY	29	2,82	61	2,81
4600	P2	LY	43	2,71	111	3,31
5642	P2	LY	18	2,00	95	2,94
5681	P2	LY	11	1,33	104	3,11
5706	P2	LY	64	2,84	91	3,34
5724	P2	LY	70	3,31	134	3,59
5732	P2	LY	76	3,48	102	3,17
1624	P3	C	39	1,92	87	2,89
3651	P3	C	48	1,54	49	2,06
4155	P3	C	41	1,57	65	2,84
4601	P3	C	22	1,54	82	2,73
4624	P3	C	42	1,57	55	2,05
5657	P3	C	32	1,75	67	2,87
5683	P3	C	43	1,31	49	2,33
5702	P3	C	33	1,82	71	2,36
5741	P3	C	45	1,44	102	3,50
2158	P3	LY	34	2,31	71	2,80
2168	P3	LY	26	1,16	57	2,10
3180	P3	LY	45	2,05	74	3,07
4600	P3	LY	23	1,73	55	2,99
5642	P3	LY	44	2,46	77	2,60
5681	P3	LY	65	2,97	86	3,14
5706	P3	LY	55	2,42	67	3,24
5732	P3	LY	57	2,14	106	3,13

(b) Milk

			Fungi		Bacteria	
Cow	Period	Group	Observed	Shannon	Observed	Shannon
1624	P1	C	17	1,99	52	2,54
2151	P1	C	23	2,47	95	3,18
3157	P1	C	9	1,03	99	3,55
3651	P1	C	24	2,37	45	3,31
4154	P1	C	7	1,01	125	3,45
4155	P1	C	10	1,49	68	3,05
4172	P1	C	9	0,83	25	0,49
4601	P1	C	13	1,93	68	2,89
4624	P1	C	12	1,75	84	3,20
5611	P1	C	9	0,61	46	2,81
5617	P1	C	9	1,49	40	1,42

5657	P1	C	16	2,34	58	2,64
5683	P1	C	10	1,08	74	2,94
5689	P1	C	8	0,04	60	2,40
5693	P1	C	7	0,74	80	3,04
5702	P1	C	4	1,03	95	3,33
5739	P1	C	11	1,46	70	2,80
5741	P1	C	8	1,54	87	3,12
2158	P1	LY	18	1,87	34	1,74
2168	P1	LY	6	0,03	74	2,58
2635	P1	LY	20	2,73	77	3,02
3180	P1	LY	10	0,92	69	2,63
3650	P1	LY	8	0,74	75	2,69
4160	P1	LY	1	0,00	49	2,24
4167	P1	LY	11	1,47	64	2,89
4168	P1	LY	9	1,54	92	2,96
4600	P1	LY	15	2,17	87	2,98
5607	P1	LY	3	0,39	58	2,30
5618	P1	LY	7	1,00	86	2,93
5635	P1	LY	12	2,08	93	2,94
5638	P1	LY	18	2,02	67	2,56
5642	P1	LY	6	1,34	57	1,84
5681	P1	LY	13	1,85	66	3,40
5706	P1	LY	17	2,43	116	3,57
5722	P1	LY	7	1,07	55	2,32
5724	P1	LY	6	1,28	89	3,18
5732	P1	LY	11	1,86	75	2,45
5754	P1	LY	12	1,08	80	3,00
1624	P2	C	22	1,27	51	1,33
2151	P2	C	17	0,63	86	3,29
3157	P2	C	20	0,47	84	3,30
3651	P2	C	22	1,13	51	2,19
4154	P2	C	15	1,15	43	3,17
4155	P2	C	13	1,57	97	2,84
4172	P2	C	15	1,02	79	2,84
4601	P2	C	20	2,17	58	2,21
4624	P2	C	22	1,89	74	2,04
5611	P2	C	15	1,07	93	3,39
5617	P2	C	1	0,00	50	2,52
5657	P2	C	4	0,40	45	1,69
5683	P2	C	12	1,32	57	1,15
5689	P2	C	15	1,60	81	3,08
5690	P2	C	14	0,58	74	3,16
5702	P2	C	13	1,98	78	3,26
5739	P2	C	3	0,87	71	3,53

5741	P2	C	12	1,63	54	2,08
2158	P2	LY	20	1,74	54	1,75
2168	P2	LY	16	0,89	62	2,68
2635	P2	LY	19	1,35	79	2,92
3180	P2	LY	3	0,89		
3650	P2	LY	12	0,53	37	2,80
4160	P2	LY	6	0,08	48	1,96
4167	P2	LY	10	1,40	86	3,28
4600	P2	LY	13	1,33	78	2,98
5607	P2	LY	9	0,51	84	3,27
5618	P2	LY	17	1,28	61	2,33
5635	P2	LY	16	1,04	119	3,11
5638	P2	LY	17	1,65	65	3,19
5642	P2	LY	19	0,57	55	1,65
5681	P2	LY	24	1,43	83	2,29
5706	P2	LY	30	0,96	88	3,01
5722	P2	LY	15	1,33	78	3,32
5724	P2	LY	20	1,47	42	2,96
5732	P2	LY	17	1,27	48	2,93
5754	P2	LY	20	2,00	90	3,11
1624	P3	C	14	0,37	51	2,14
3157	P3	C	4	0,95	74	2,53
3651	P3	C	4	0,01	40	2,45
41-5	P3	C	16	0,61		
4601	P3	C	3	0,01	27	1,27
5657	P3	C	10	1,44	62	1,74
5683	P3	C	4	0,34	58	2,46
5689	P3	C	4	0,03	40	1,44
5693	P3	C	3	0,10	70	2,89
5739	P3	C	11	0,56	40	2,17
5741	P3	C	11	0,24	98	3,01
2158	P3	LY	14	0,31	28	0,98
2168	P3	LY	14	1,17	51	2,16
2635	P3	LY	12	1,10	64	2,76
3650	P3	LY	9	0,07	52	2,19
4160	P3	LY	3	0,01	36	2,02
4167	P3	LY	4	0,01	46	1,88
4600	P3	LY	6	0,63	76	3,46
5607	P3	LY	4	0,71	36	2,00
5618	P3	LY	3	0,01	70	2,94
5681	P3	LY	1	0,00	84	3,21
5706	P3	LY	12	1,22	85	2,35
5722	P3	LY	5	0,25	49	2,81
5724	P3	LY	9	0,14	31	1,90

5732	P3	LY	12	1,19	40	1,53
5754	P3	LY	6	0,04	46	2,27
5690	P1	C			64	2,62
5693	P2	C			29	1,79
4168	P2	LY			58	2,98
2151	P3	C			65	2,92
4154	P3	C			23	2,29
4155	P3	C			33	2,77
4172	P3	C			59	1,82
4624	P3	C			52	2,11
5611	P3	C			36	2,46
5617	P3	C			31	1,17
5690	P3	C			33	2,47
5702	P3	C			47	2,57
3180	P3	LY			25	1,47
4168	P3	LY			44	2,29
5635	P3	LY			17	0,90
5638	P3	LY			58	2,81
5642	P3	LY			36	1,68

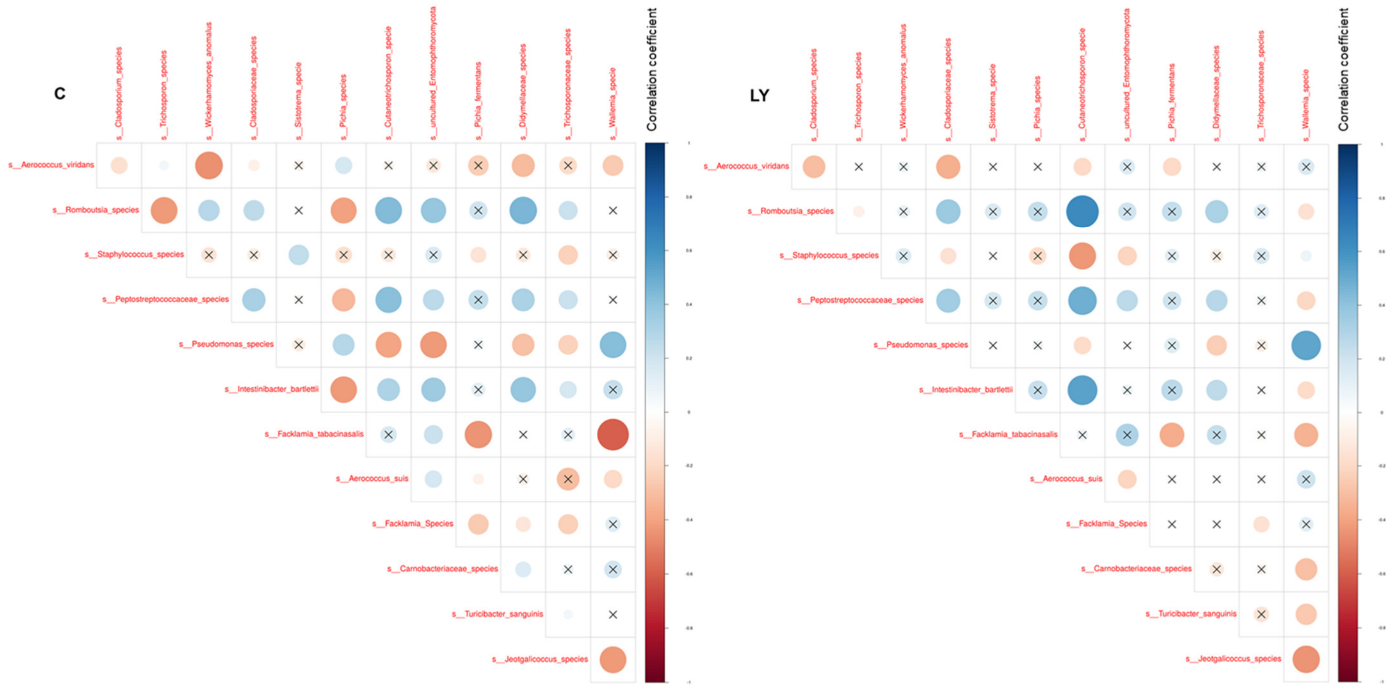

Figure S1. Correlogram of bacterial and fungal taxa with more than 1% of abundance in teat suspensions from the C and LY groups. Color intensity and the size of the circle are proportional to the **correlation coefficients**: blue color = positive correlations; red color = negative correlations. Crosses (x) indicate insignificant correlations (p-value > 0.05).
